# Genes conserved in bilaterians but jointly lost with Myc during nematode evolution are enriched in cell proliferation and cell migration functions

**DOI:** 10.1007/s00427-015-0508-1

**Published:** 2015-07-15

**Authors:** Albert J. Erives

**Affiliations:** Department of Biology, University of Iowa, Iowa City, IA 52242-1324 USA

**Keywords:** Cancer, Cell proliferation, Cell migration, Apoptosis, Myc, Mnt, Myc synthetic lethals (MycSL genes), Genetic screens, Comparative genomics

## Abstract

Animals use a stereotypical set of developmental genes to build body architectures of varying sizes and organizational complexity. Some genes are critical to developmental patterning, while other genes are important to physiological control of growth. However, growth regulator genes may not be as important in small-bodied “micro-metazoans” such as nematodes. Nematodes use a simplified developmental strategy of lineage-based cell fate specifications to produce an adult bilaterian body composed of a few hundreds of cells. Nematodes also lost the *MYC* proto-oncogenic regulator of cell proliferation. To identify additional regulators of cell proliferation that were lost with *MYC*, we computationally screened and determined 839 high-confidence genes that are *conserved in bilaterians/lost in nematodes* (CIBLIN genes). We find that 30 % of all CIBLIN genes encode transcriptional regulators of cell proliferation, epithelial-to-mesenchyme transitions, and other processes. Over 50 % of CIBLIN genes are unnamed genes in *Drosophila*, suggesting that there are many understudied genes. Interestingly, CIBLIN genes include many Myc synthetic lethal (MycSL) hits from recent screens. CIBLIN genes include key regulators of heparan sulfate proteoglycan (HSPG) sulfation patterns, and lysyl oxidases involved in cross-linking and modification of the extracellular matrix (ECM). These genes and others suggest the CIBLIN repertoire services critical functions in ECM remodeling and cell migration in large-bodied bilaterians. Correspondingly, CIBLIN genes are co-expressed with Myc in cancer transcriptomes, and include a preponderance of known determinants of cancer progression and tumor aggression. We propose that CIBLIN gene research can improve our understanding of regulatory control of cellular growth in metazoans.

**Electronic supplementary material:**

The online version of this article (doi:10.1007/s00427-015-0508-1) contains supplementary material, which is available to authorized users.

## Introduction

Genetic screens have been successful in identifying developmental regulators of patterning, axis formation, and cell fate specification in animals and most of these are both conserved across and specific to animals (Chen et al. 1996; Haffter et al. 1996; Kane et al. 1996; Kelsh et al. 1996; Mullins et al. 1994; Mullins et al. 1996; Mullins and Nusslein-Volhard 1993; Nusslein-Volhard and Wieschaus 1980). A clinical approach accelerated by genomic and transcriptomic studies of diseased tissues has also identified key developmental regulators of cell growth and replication, many of which must be more easily identified when mutated in a restricted population of somatic cells contributing to tumors and metastatic cancers (Ben-Porath et al. 2008; Beroukhim et al. 2010; Cancer Genome Atlas Research Network 2008; Dalgliesh et al. 2010; Ding et al. 2008; Jones et al. 2008; Parsons et al. 2008; Pece et al. 2010; Pleasance et al. 2010). Genetic screens based on short hairpin RNAs (shRNAs) and small interfering RNAs (siRNAs) have emerged as technological improvements to mutational screens (Berns et al. 2004; Moffat et al. 2006; Ngo et al. 2006; Paddison et al. 2004). Multiplexing these screens with a library of diverse expression drivers (i.e., libraries of different regulatory DNAs) would make these approaches more systematic. However, such regulatory multiplexing is limited by the coverage or complexity of current expression libraries, as well as the burden of needing redundancy in the library to reduce the number of false-positives. Thus, a complete expression library by shRNA library screen would be too prohibitive in terms of time and resources required. Thus, until these challenges are addressed, shRNA/siRNA screens are most productive with carefully designed synthetic lethal or synthetic sick phenotype as has been done around the Myc proto-oncogene frequently amplified in many cancers (Cermelli et al. 2014; Kessler et al. 2012; Toyoshima et al. 2012).

Well-designed shRNA/siRNA genetic screens sometimes rely on a synthetic phenotype caused by abnormal interactions with a previously known regulator. To address the limited scope of such synthetic screens, one could make use of the multiple sequenced genomes now available for entire phyla and subclades of animals. A comparative genomics approach would be unaffected by issues of genetic penetrance, tissue specificity, lethality, and might be more open-ended. Furthermore, a robust comparative “systems genetics” approach emerges when comparative genomics is coupled with transcriptomic, proteomic, and subcellular localization data. Using this approach, we recently identified genes that are conserved in eukaryotes but missing in animals (Erives and Fassler 2015). We refer to these as *conserved in eukaryotes/lost in animals* (CIELIM) genes. Analysis of the CIELIM repertoire shows that they encode many chaperones and amyloid disaggregases (Hsp78, Hsp104, and New1/EF3) and that their loss in animals could be connected to the loss of many of their client metabolic enzymes (Erives and Fassler 2015). This study resolved what has been described as the baffling absence of Hsp104 in Metazoa, where it could play a role in ameliorating many types of polyglutamine-induced neural degeneration disorders if it were only present (Cushman-Nick et al. 2013). Thus, the comparative systems genetics approach can be exploited in unprecedented ways to identify cohorts of genes associated with specific disease processes in unexpected ways.

Here, we apply the comparative systems genetic approach to cancer by identifying *conserved in bilaterians/lost in nematodes* (CIBLIN) genes. The basis for this computational genomic screen is that nematodes represent a derived bilaterian phylum characterized by an evolutionary reduction in body size and adoption of cell fate determinative mechanisms based on short, well-defined cell division lineages, the majority of which are homologous across the phylum (Houthoofd et al. 2003; Schierenberg 2006). Furthermore, at the completion of nematode embryogenesis, cellular divisions cease in all somatic organs and tissues (Hyman 1940). The number of somatic cells (or nuclei in the case of syncytial tissues) is subsequently held constant throughout the adult worm’s life span without cellular replenishment. For example, *Caenorhabditis elegans* is a typical nematode from the class Chromadorea, and as an adult body is composed of only 959 somatic cells. This extremely small bodied lifestyle and reliance on cell fate determinative mechanisms based on cell lineage is an evolutionary derived condition that is atypical of most bilaterians, including deuterostomes (e.g., humans, ascidians, and echinoderms) and protostomes from either Ecdysozoa (e.g., fly and nematode) or Lophotrochozoa (e.g., molluscs and polychaetes). Even other clades that have independently evolved cell fate determinative mechanisms based on short cell lineages (e.g., the ascidian tadpole) still build and pattern relatively large bodied adults after metamorphosis using cell signaling induction mechanisms to exert control of local cell proliferation and differentiation (Satoh 1994). Nonetheless, almost all major developmental pathways (e.g., EGF, FGF, Hedgehog, Notch, and Wnt pathways) are maintained in nematodes (Kolotuev et al. 2009; Minor et al. 2013; Schmid and Hajnal 2015). Furthermore, even components of the Hippo pathway, which is intimately connected to regulation of organ size, are mostly conserved in nematodes (Yang and Hata 2013).

In contrast, *C. elegans* and all other nematodes lost the *MYC* proto-oncogene despite its astonishing conservation in all other animals and their closest non-animal relatives (Brown et al. 2008; Young et al. 2011). The role of Myc in cell proliferation can be understood in part by its regulation of ribosome biogenesis genes, but it has so many effects that its exact role is still debated (Brown et al. 2008; Grewal et al. 2005). The loss of the *MYC* gene in the small-bodied nematodes indicates that they may have lost many other such regulators of cell proliferation and related processes absent the need to surveil, control, and coordinate large populations of cells in bulky tissues and organs. These losses would have evolved alongside increasing developmental reliance on determinate cell lineage specifications. In principle, diverse nematode genomes can be exploited to identify genes that are CIBLIN genes. Thus, CIBLIN regulators would include Myc and perhaps many other important developmental regulators of cell proliferation.

Here, we show that there are 839 human CIBLIN orthology groups with homologs in the genomes of mouse, fly (*Drosophila melanogaster*), and beetle (*Triboleum castaneum*) but not in nematode genomes corresponding to five *Caenorhabditis* species (*C. elegans* Sequencing Consortium 1998; Stein et al. 2003), *Pristionchus pacificus* (Dieterich et al. 2008), *Brugia malayi* (Ghedin et al. 2007), *Loa loa* (Desjardins et al. 2013), *Onchocerca volvulus* (Desjardins et al. 2013; Unnasch and Williams 2000), or the enoplean nematode *Trichinella spiralis* (Mitreva et al. 2011), which serves as an outgroup to all the other nematodes. These genes are overwhelmingly associated with developmental processes and encode transcriptional regulators and other signaling proteins. The list of CIBLIN genes includes many hits from different Myc synthetic lethal screens. Furthermore, we find that CIBLIN genes include many regulators of cell proliferation, epithelial-to-mesenchyme transition, and cell migration. Last, CIBLIN regulators are co-expressed with *MYC* in cancer transcriptomes, and many have already been identified as drivers and/or markers of aggressive cancer types. These findings validate the comparative genomic “screening” approach as a robust and efficient complement to conducting new genetic screens based on synthetic phenotypes in select tissues and cells. We propose that the CIBLIN repertoire constitutes the core proto-oncogenic genomic compartment targeted during human cancer progression. Furthermore, the joint loss of the CIBLIN genes in nematodes suggests that these genes should be studied concurrently to identify the ways in which they interact with each other in organogenesis, in the surveillance and maintenance of cell number, and in the attenuation of disease.

## Materials and methods

### Comparative genomic analyses.

Orthology relationships were determined by iterative cross-checking between species using EnsemblCompara data (Vilella et al. 2009) and the BioMart database query and filtering tool (Guberman et al. 2011; Haider et al. 2009) using Excel spreadsheets and conditional sorting to collect a desired homology class (e.g., highlighting and keeping only the “one-to-one” homology calls) before retrieving the next set of genes based on the Ensembl gene IDs and filtering for protein-coding genes (Table 1). This strategy was used previously to identify the CIELIM genes (Erives and Fassler 2015). CIBLIN orthology relationships were re-rechecked for nematodes in both the Metazoa Ensembl data sets and the Ensembl Genes 78 data sets (Birney et al. 2004; Vilella et al. 2009) as indicated in Table 1 and for reasons described in the text. Omega (dN/dS) values were obtained via BioMart retrieval of the human, mouse, and rat values relative to human Ensembl gene IDs.

**Table 1:** The 839 *conserved in bilaterians/lost in nematodes* (CIBLIN) genes

Set^a^	Orthology groups checked and identified in:	Orthologs NOT called in:	Data system	No. of distinct fly genes	No. of distinct human genes
1	*Drosophila melanogaster*, *Tribolium castaneum*	*Caenorhabditis brenneri*, *C. briggsae*, *C. elegans*, *C. japonica*, *C. remanei*, *Brugia malayi*, *Loa loa*, *Onchocerca volvulus*, *Pristionchus pacificus, Trichinella spiralis*	Metazoa EnsemblCompara (invertebrates)	3009	n.d.
2	*D. melanogaster*, *H. sapiens*, *M. musculus* ^b^ (839 orthology groups)	*C. elegans*	Ensembl Genes 78 (vertebrates + *D. melanogaster*, *C. elegans*, *S. cerevisiae*)	1197	1389
3	*D. melanogaster*	*S. cerevisiae*	Ensembl Genes 78	968	1158
4	Three-way strict orthology across *H. sapiens*, *M. musculus*, and *R. norvegicus*	N/A	Ensembl Genes 78	881	971

Gene sets identified and analyzed in this study. Rows in yellow represent three CIBLIN gene lists. Set 1 is from a precursor step. Set 2 includes all CIBLIN genes, including those duplicated in any one lineage and regardless of whether they are present outside of Bilateria. Other sets are used for specific analyses described in the text

*n.d.* not determined, *N/A* not applicable

^a^Each numbered set describes a subset of genes identified from the previous set

^b^Human-mouse many-to-many relationships (independent duplications) were removed. This predominantly removes multi-copy genes such as histone-encoding genes

### Phylogenetic analyses.

**Fig. 1:**
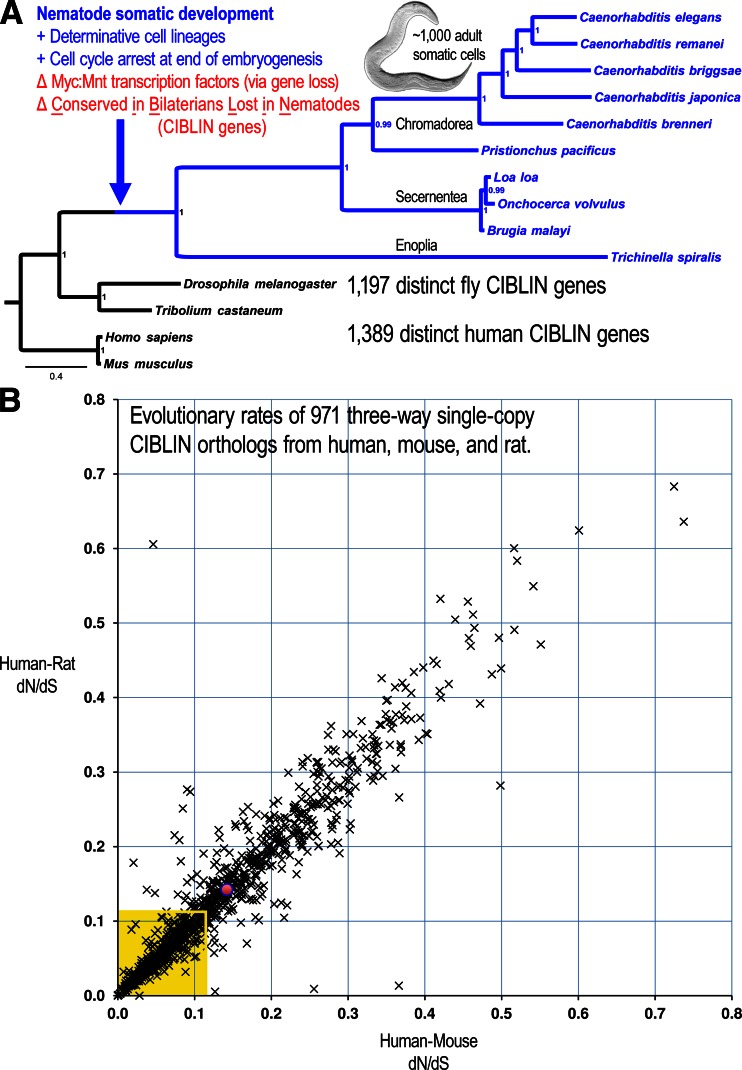
Identification of CIBLIN genes. **a** Sufficient genomes and comparative genomic resources exist to attempt a screen for genes *conserved in bilaterians/lost in nematodes* (CIBLIN). Tree is based on the phylogenetic analysis of the Med12 protein sequence, which is not lost (see “Materials and methods”). Tree shows only the species whose genomes were used to search for genes lost in the stem-nematode lineage, during which the genes encoding the Myc and Mnt bHLH transcription factors were lost. The identification of genes lost in the stem-nematode lineage might correspond to general cell proliferation programs used by animals. Image of nematode is of an adult *C. elegans*, which has only 959 somatic cells in the adult (image adapted from Bob Goldstein, UNC Chapel Hill, CC-BY-A 2006). **b** Plot of evolutionary rates for 971 CIBLIN orthologs present as single-copy genes in mammals. Graph plots each gene using the ω values (*dN*/*dS*) computed between the human and mouse genes (*x*-axis) or the human and rat genes (*y*-axis), and shows that these genes predominantly evolve at clock-like rates, indicating negative (purifying) selection. The *red dot* represents the average rates for the 971 mammalian CIBLIN genes (~0.14) indicating that most of these are diverging only slowly. The *box in yellow* encloses the most conserved ~490 mammalian CIBLIN genes, which correspond to the ranked set at which “developmental process” is most significant of all ranked sets (167 N genes/top 490 M genes; see Table 3). Thus, the GO attribute for “developmental process” is significantly overrepresented in the most conserved CIBLIN genes

The MUltiple Sequence Comparison by Log-Expectation (MUSCLE) alignment algorithm and MEGA6 were used to generate alignments of the Med12 and Med15/Mdt15 sequences (Edgar 2004a; Edgar 2004b; Tamura et al. 2013). Phylogenetic analysis was conducted using Bayesian MCMC, and mixed amino acid models were tested via MrBayes (Huelsenbeck and Ronquist 2001; Ronquist and Huelsenbeck 2003; Ronquist et al. 2012). Sufficient generations were run for the average standard deviation of split runs to be less than 1 %. The numbers on the nodes in the tree in Fig. 1 represent posterior probabilities.

### GO correlations.

Gene Ontology (GO) attribution term enrichment analysis was conducted using FuncAssociate 2.1 using ordered and unordered rankings as described in the text (Berriz et al. 2009; Berriz et al. 2003). Some GO term results were removed from the tables shown if they were redundant with other terms on the list in terms of attribution name and in terms of gene identities. Some terms are also abbreviated so that they fit in the tables. Human Ensembl gene IDs were used for the name space for all GO analyses (see Tables S1, S2, S3, and S4).

### Interactome and gene co-expression analyses.

**Fig. 2:**
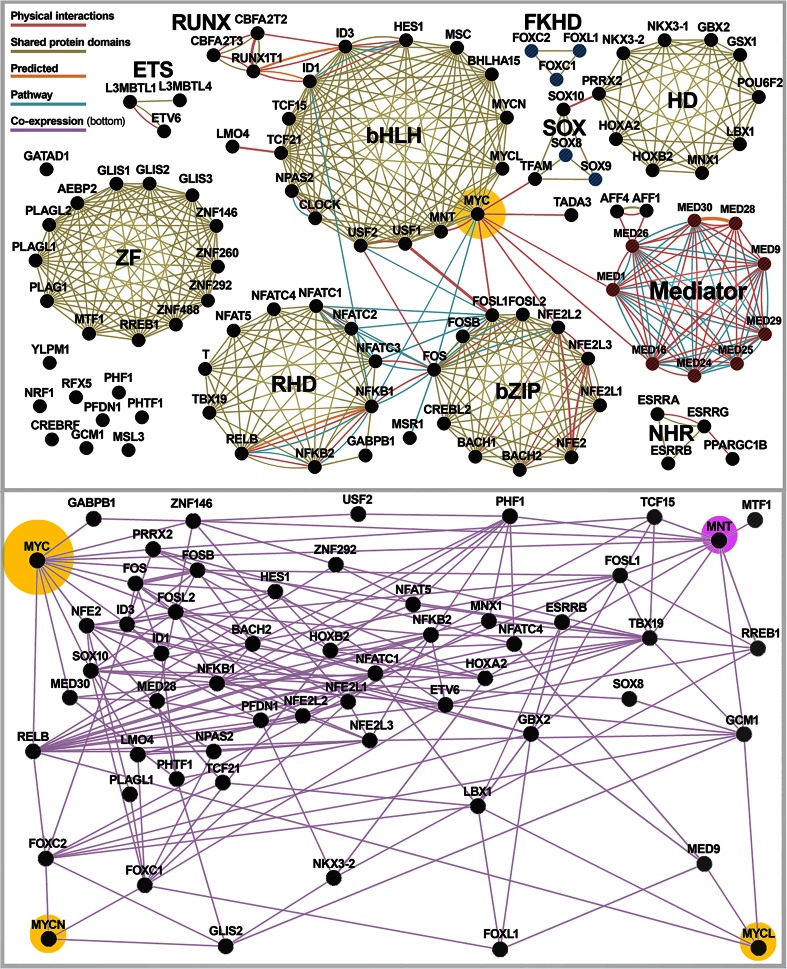
Interaction network for human CIBLIN transcriptional regulators. **a** Of the 1158 human CIBLIN genes (set 3, Table 1), 101 have GO attributes associated with either “*sequence-specific DNA binding transcription factor activity*” (GO:0003700) or “*Mediator complex*” (GO:0016592). The *top panel* shows the interaction network for the human genes based on physical interaction interactome data, shared protein domains, predicted based on other species (e.g., studies in mouse and others); and pathway interactome. The *bottom panel* shows a subset of 56 genes that are most closely expressed with *MYC* (*big yellow halo*), *MYCN* (*small yellow halo*), *MYCL* (*small yellow halo*), or *MNT* (*small pink halo*) based on all available human transcriptome studies. The percent contribution of each study to the expression association map is predominantly associated with cancer transcriptomes (see Table 4). **b** Co-expression network for 52 regulator genes (a subset of genes in Fig. 2a) co-expressed with *MYC*, *MYCN*, *MYCL*, and *MNT* (highlighted gene nodes in each corner) over 287 transcriptomic studies using human cells. The specific studies that contributed the most to the Pearson correlations between these genes are listed in Table S5 and ranked by weight

The GeneMania system was used to identify network interactions using the associated human gene names (Montojo et al. 2014; Mostafavi et al. 2008; Warde-Farley et al. 2010; Zuberi et al. 2013). The GeneMania analysis of Fig. 2a was restricted to the 101 input genes identified via BioMart filter (Guberman et al. 2011; Haider et al. 2009) using the GO attribution indexes GO:0003700 (sequence-specific DNA binding transcription factor activity) or GO:0016592 (Mediator complex). The following data types, but not all of these, are shown for better clarity: physical interaction, shared protein domains, predicted, pathway, co-expression, and co-localization. The GeneMania analysis of Fig. 2b was restricted to the 56 input genes shown and the co-expression datasets based on the genes having direct connections to MYC, MYCN, MYCL, MNT, or at least two or more connections to these genes and direct connections. The transcriptomic studies out of 287 available meta-studies that contributed the most to the correlations of this set of genes are ranked by weight in Supplementary Table S5.

## Results

### An upper-bound of 839 CIBLIN orthology groups, including MYC and MNT

We use the term “orthologs” to include genes that may have duplicated in any one single lineage, but not homologous genes that are members of more distantly-related paralogy groups established prior to bilaterian diversification. To identify and/or enrich for CIBLIN genes (conserved in bilaterians, lost in nematodes), we took the approach of identifying orthologs conserved in two mammalian deuterostomes (human and mouse) and two insect protostomes (fly *D. melanogaster* and the beetle *T. castaneum*). We did not see the justification for using additional bilaterian gene sets from non-nematode genomes for the following two reasons. First, both insects and nematodes are ecydozoan protostomes and this gave us representatives in a clade that encompasses the desired gene losses. Second, we did not want to miss genes because of incomplete assemblies and these four genomes have been assembled to near completion more so relative to other genomes. At the same time, we eliminated genes computed to be orthologs in any nematode genome for which orthology data has been computed genome-wide for a set of genomes. Thus, while we kept bilaterian orthologs that are present in the four non-nematode genomes (human, mouse, fly, beetle), we eliminated all orthologs if they were called even once in any one of ten diverse nematode genomes (see phylogenetic tree in Fig. 1a, which is based on Med12, which is a conserved co-activator subunit of Mediator that is not lost in nematodes). Thus, our list of CIBLIN genes represents genes that truly have been lost early in nematode evolution or else have diverged so far that they are not reliably detectable with confidence in any one of the examined nematode genomes.

We began with the maximum likelihood (ML)-based EnsemblCompara orthology calling data computed for invertebrate genomes to identify all orthologs present in fly and beetle but absent in the ten nematode genomes (gene set #1 in Table 1, and “Materials and methods”). Once sorted, these genes amount to 3009 unique genes in *Drosophila*. We next took these fly genes and identified the subset with orthologs in the human and mouse genomes. This second step was conducted using the orthology calls for Ensembl Genes 78, which is based on the application of the same Ensembl ML pipeline applied to a large set of vertebrate genomes along with just a few genomes from model genetic systems (fly, worm, yeast). Because orthology determination on such large data sets are influenced by the genomes used, we also removed a few genes at this step that were called as orthologs to *C. elegans* in the Ensembl Genes 78 Compara data. This second step produced the CIBLIN genes corresponding to 1197 unique fly genes and 1389 unique human genes (gene set #2 in Table 1 and Supplementary Table S1). These 1389 human genes correspond to 496 genes maintained as single-copy genes in both humans and flies, 231 orthology groups that duplicated in the human lineage specifically, 58 orthology groups that duplicated in the fly lineage specifically, and 54 genes that duplicated in both human and fly lineages (Supplementary Table S1). These genes correspond to only 839 different orthology groups in humans.

To understand the difference between metazoan-specific CIBLIN genes and more ancient eukaryotic CIBLIN genes, we also produced a third set of genes that are not computed to have orthologs in the yeast *S. cerevisiae*. However, this removed only 229 fly genes, which were orthologous to 178 yeast genes, leaving 1158 human genes and 968 fly genes (gene set #3 in Table 1 and Supplementary Table S2).

To better understand the evolutionary rates of the CIBLIN genes, we identified 971 human CIBLIN genes that are maintained as single-copy genes in both the mouse and rat genomes (gene set #4 in Table 1 and Supplementary Tables S3 and S4), and plotted the omega (ω) ratios (dN/dS) for both the human/mouse and human/rat alignments of each gene (Fig. 1b). This is a ratio of the number of nonsynonymous substitutions per non-synonymous site (*dN*) to the number of synonymous substitutions per synonymous site (*dS*). If the rat and mouse ω values are quite different, this would indicate that this gene is either under positive or relaxed selection in one lineage, or else is poorly annotated in one genome. This plot shows that the majority of CIBLIN genes are under extreme negative or purifying selection (avg. ω is ~0.14 for both rat and mouse genes relative to humans) and are evolving slowly at similar clock-like rates in each rodent genome (see majority of points along identity line in Fig. 1b).

### CIBLIN genes encode regulators of transcription, cell proliferation, and cell migration

To identify the types of biological functions associated with the CIBLIN gene repertoire, we conducted an analysis for statistically significant enrichment of GO attributions (see “Materials and methods”). We first took the single-copy mammalian CIBLIN genes and conducted a test on an unordered list of the 971 human genes. Four of the ten total GO terms were for similar functions connected to transcriptional regulation: “regulation of gene expression,” “transcription from RNA polymerase II promoter,” “sequence-specific DNA binding transcription factor activity,” and “nucleic acid binding transcription factor activity” (Table 2). These four terms were associated with 299 genes, which represent ~31 % of the 971 genes. Thus, 1/3 of the CIBLIN genes are transcriptional regulators.

**Table 2:** Transcriptional regulators are overrepresented in the 971 mammalian CIBLIN orthologs

*N*	X	*P*	*P*_adj_	Attrib. ID	Gene Ontology (GO) attribution name
16	53	3.3E−09	<0.001	GO:0008146	Sulfotransferase activity
17	64	9.4E−09	<0.001	GO:0016782	Transferase act., transferring sulfur-containing groups
259	4010	1.7E−06	0.004	GO:0010468	Regulation of gene expression
45	431	2.3E−06	0.005	GO:0006366	Transcription from RNA polymerase II promoter
85	1025	2.5E−06	0.005	GO:0003700	Sequence-specific DNA binding transcription factor act.
85	1026	2.6E−06	0.006	GO:0001071	Nucleic acid binding transcription factor activity
4	4	6.1E−06	0.022	GO:0004720	Protein-lysine 6-oxidase activity
6	12	1.1E−05	0.032	GO:0048484	Enteric nervous system development
6	12	1.1E−05	0.032	GO:0070286	Axonemal dynein complex assembly
7	18	1.5E−05	0.040	GO:0001539	Cilium or flagellum-dependent cell motility

Functions associated with cell migration or remodeling of extra cellular matrix are highlighted in green. Functions associated with transcriptional regulation are highlighted in red and correspond to exactly 299 genes with these terms (~1/3 of the genes). Functions associated with development are highlighted in yellow. For context, the human Gene Ontology database is composed 19,452 genes with 17,658 attributes

*N* number of genes in the tested set that match the number of genes with the given GO Attribution. *X* the total number of genes in the genome with that attribute, *P* the “single hypothesis one-sided *P* value of the association between attribute and query based on Fisher’s exact test” (Berriz et al. 2009; Berriz et al. 2003), *P*_*adj*_ an empirically adjusted *P* value, which is the “fraction of 1000 null-hypothesis simulations having attributes with this single-hypothesis *P* value or smaller” (Berriz et al. 2009; Berriz et al. 2003)

Remarkably, five of the six remaining significantly enriched GO terms correspond to three distinct functions, all of which are connected to cell migration and/or the remodeling of the extracellular matrix (ECM). First, 17 genes were connected to sulfotransferase activities, many of which are known to be important in regulating the interactions between tumorous cells and their microenvironment via their control of sulfation patterns on heparan sulfate proteoglycans (HSPGs) in the ECM (Solari et al. 2014). Second, all of the genes encoding the LOX, LOX2, LOX3, and LOX4 enzymes appear to be missing. These are secreted by tumors and are involved in cancer progression through their role in the post-translational oxidative deamination of peptidyl lysine residues on fibrous collagen and elastin (Barker et al. 2012). Last, seven of the human CIBLIN genes encode highly conserved orthologs within the dynein heavy and light chain families involved in “cilium or flagellum-dependent cell motility.” Thus, CIBLIN genes predominantly contain transcriptional regulators, as well as important enzymatic systems and cellular components responsible for cell migration and ECM remodeling.

### The most conserved CIBLIN genes encode transcriptional regulators of development

To identify the functions of the most conserved 971 mammalian CIBLIN genes, we ran a GO enrichment analysis configured to consider the ranked order at which a term is the most significant using the average ω values to order genes from slowest to fastest evolving (among mammals). This alternate analysis would identify significant GO terms associated with the genes under the greatest amount of purifying selection. This second GO analysis shows that the vast majority of enriched terms are associated with gene regulation and the control of developmental processes such as “embryonic morphogenesis,” “organ morphogenesis,” “tissue development,” “positive regulation of stem cell proliferation,” and “regulation of multicellular organism development,” validating the basic CIBLIN screen premise (Table 3). Thus, for example, the term for “developmental process” is most significant when the first 489 genes are considered (i.e., the 489 most conserved genes in the 971 list based on divergence rates in rodents). Of these 489 top genes, 167 genes, or ~34.1 %, are connected to developmental processes. Various terms connected to transcriptional regulation continue to be overrepresented (Table 3).

**Table 3:** Developmental regulatory processes are most overrepresented in the most conserved CIBLIN orthologs

*N*	M^a^	X	*P*	*P*_adj_	Attrib. ID	Gene Ontology (GO) attribution name
13	66	384	4.2E−10	0.000	GO:0048598	Embryonic morphogenesis
16	785	53	1.5E−10	0.000	GO:0008146	Sulfotransferase activity
17	785	64	3.6E−10	0.000	GO:0016782	Transferase act., transferring sulfur-con. groups
18	136	407	4.8E−10	0.000	GO:0009887	Organ morphogenesis
15	260	179	1.8E−08	0.000	GO:0030278	Regulation of ossification
27	248	550	2.1E−09	0.000	GO:0009888	Tissue development
27	212	666	3.9E−09	0.000	GO:0048731	System development
49	128	2675	3.2E−12	0.000	GO:0048856	Anatomical structure development
41	204	1273	7.7E−11	0.000	GO:0009653	Anatomical structure morphogenesis
39	320	789	8.0E−10	0.000	GO:0043565	Sequence-specific DNA binding
50	298	1290	9.3E−10	0.000	GO:0045595	Regulation of cell differentiation
55	407	1025	1.1E−10	0.000	GO:0003700	Sequence-specific DNA binding txn. factor activity
55	407	1026	1.1E−10	0.000	GO:0001071	Nucleic acid binding transcription factor activity
59	318	1502	1.8E−10	0.000	GO:0006357	Reg. of txn. from RNA polymerase II promoter
69	343	1778	2.2E−10	0.000	GO:0050793	Regulation of developmental process
100	320	3294	1.3E−10	0.000	GO:0006355	Regulation of transcription, DNA-templated
101	320	3424	5.1E−10	0.000	GO:0051252	Regulation of RNA metabolic process
108	320	3716	2.2E−10	0.000	GO:0010556	Reg. of macromolecule biosynthetic process
110	320	3928	1.3E−09	0.000	GO:0009889	Regulation of biosynthetic process
91	433	2234	5.0E−09	0.000	GO:0048869	Cellular developmental process
108	320	3895	3.5E−09	0.000	GO:0031326	Regulation of cellular biosynthetic process
139	411	4010	1.5E−10	0.000	GO:0010468	Regulation of gene expression
156	488	3994	9.0E−10	0.000	GO:0044767	Single-organism developmental process
167	489	4456	4.6E−09	0.000	GO:0032502	Developmental process
35	559	431	3.3E−08	0.001	GO:0006366	Transcription from RNA polymerase II promoter
153	411	4917	3.0E−08	0.001	GO:0060255	Regulation of macromolecule metabolic process
8	305	34	4.1E−08	0.002	GO:0032570	Response to progesterone
47	318	1230	4.0E−08	0.002	GO:1902680	Positive regulation of RNA biosynthetic process
48	318	1273	4.3E−08	0.002	GO:0010628	Positive regulation of gene expression
9	128	115	6.5E−08	0.003	GO:0061448	Connective tissue development
26	316	474	6.5E−08	0.003	GO:0008134	Transcription factor binding
42	387	861	7.2E−08	0.003	GO:0045944	Pos. reg. of txn. from RNA pol. II promoter
45	318	1172	7.1E−08	0.003	GO:0045893	Pos. regulation of transcription, DNA-templated
47	318	1251	6.7E−08	0.003	GO:0051254	Positive regulation of RNA metabolic process
51	318	1405	5.1E−08	0.003	GO:0010557	Pos. reg. of macromolecule biosynthetic process
150	320	6382	6.8E−08	0.003	GO:0044260	Cellular macromolecule metabolic process
133	320	5472	1.0E−07	0.006	GO:0031323	Regulation of cellular metabolic process
5	40	60	1.4E−07	0.008	GO:2000648	Positive regulation of stem cell proliferation
46	295	1336	1.2E−07	0.008	GO:2000026	Reg. of multicellular organismal development
53	318	1534	1.3E−07	0.008	GO:0009891	Positive regulation of biosynthetic process
31	204	1062	2.0E−07	0.012	GO:0048513	Organ development
35	311	840	2.1E−07	0.012	GO:0051094	Positive regulation of developmental process

Gene Ontology (GO) attributes related to developmental processes, stem cell proliferation, and organogenesis are predominantly associated with the CIBLIN repertoire (yellow highlighted rows). GO attributes related to DNA-binding transcriptional regulators are also overrepresented (light red highlighted rows.) The 167 CIBLIN genes with the GO term for developmental process (dark yellow highlight) are highlighted in Fig. 1b

*P* single hypothesis one-sided *P* value of association between attribute and query based on Fisher’s exact test, *P*_adj_ is an empirically adjusted P-value given by the fraction of 1000 null-hypothesis simulations having attributes with this single-hypothesis *P* value or smaller

^a^Genes ordered by average human/mouse and human/rat *dN*/*dS* ratios from slowest to fastest rates. In this context, M corresponds to the first M genes in the ranked list producing the most significant *P* value for any significant attribute among 17,658 attributes

Inspection of the most conserved CIBLIN genes shows that most are regulators of cell proliferation during development while others are regulators of epithelial-to-mesenchyme transitions. Many of the top conserved CIBLIN genes (e.g., *BZW1*, *LMO4*, *OTP*, *PRRX1*, *TADA3*, *MAD2L2*, *SHOX2*, *PTOV1*, and *SOX10*) have already been implicated in promoting aggressive cancers of many types (see Table 4 for references). Thus, it appears that the CIBLIN genes are predominantly a proto-oncogenic repertoire of developmental regulators.

**Table 4:** Examples of conserved CIBLIN genes with roles in development and/or cancer

RANK	Avg. *dN*/*dS* ^a^	Human gene	Human gene description [source: HGNC]	Roles in development and/or cancer progression
1	0.000	BZW1	Basic leucine zipper and W2 domains 1	Proliferation regulator, in salivary mucoepodermoid carcinoma (Li et al. 2009)
2	0.000	ENY2	Enhancer of yellow 2 homolog (*Drosophila*)	Insulator/barrier regulator, binds CTCF (Maksimenko et al. 2014)
3	0.000	LMO4	LIM domain only 4	Proliferation and epithelial-to-mesenchyme regulator; neuroblastomas; mammary stem cells and breast tumorigenesis (Ferronha et al. 2013; Salmans et al. 2014)
7	0.001	OTP	Orthopedia homeobox	Breast cancer; pulmonary carcinoids (Kim et al. 2012; Swarts et al. 2013)
11	0.006	PRRX1	Paired related homeobox 1	Gioblastoma invasiveness (Sugiyama et al. 2014); gastric cancers, regulator of epithelial-to-mesenchyme transitions (Guo et al. 2015)
14	0.008	TADA3	Transcriptional adaptor 3	Embryonic progression, cell cycle checkpoint (Mohibi et al. 2012); p53 acetylation, cellular senescence (Nag et al. 2007; Sekaric et al. 2007); cervical carcinomas, inactivation by HPV (Kumar et al. 2002)
15	0.008	MAD2L2	MAD2 mitotic arrest deficient-like 2 (yeast)	Mitotic check point (Cahill et al. 1999); chromosome instability, renal carcinomas, breast cancers, other cancers
28	0.014	SHOX2	Short stature homeobox 2	Embryoid bodies, hepatocellular carcinoma, breast cancer, lung cancers (Schneider et al. 2011)
31	0.015	PTOV1	Prostate tumor overexpressed 1	Epithelial ovarian cancers, prostate cancers, high grade malignant tumors (Alana et al. 2014)
33	0.015	GBX2	Gastrulation brain homeobox 2	Promotes pluripotent cell fates (Tai and Ying 2013)
34	0.015	SOX10	SRY-box 10	Melanoma progression (Shakhova et al. 2012)

See Supplementary tables for complete list

^a^Average *dN*/*dS* values are the average of the human/mouse and human/rat alignments

### MYC and MNT are part of a gene regulatory network of CIBLIN cell proliferation regulators

To better understand the nature of this CIBLIN regulatory repertoire, we identified 101 human CIBLIN genes having the GO attributions for either “sequence-specific DNA binding transcription factor activity” (GO:0003700) or “Mediator complex” (GO:0016592). We then used these 101 CIBLIN gene regulators in an interactome analysis to identify and rank the types of interconnections over several data types (e.g., physical interaction data sets, genetic interaction data sets, co-expression, co-localization, shared protein domain, and predicted based on interactions in orthologs of other species; see Fig. 2 and “Materials and methods”).

**Fig. 3:**
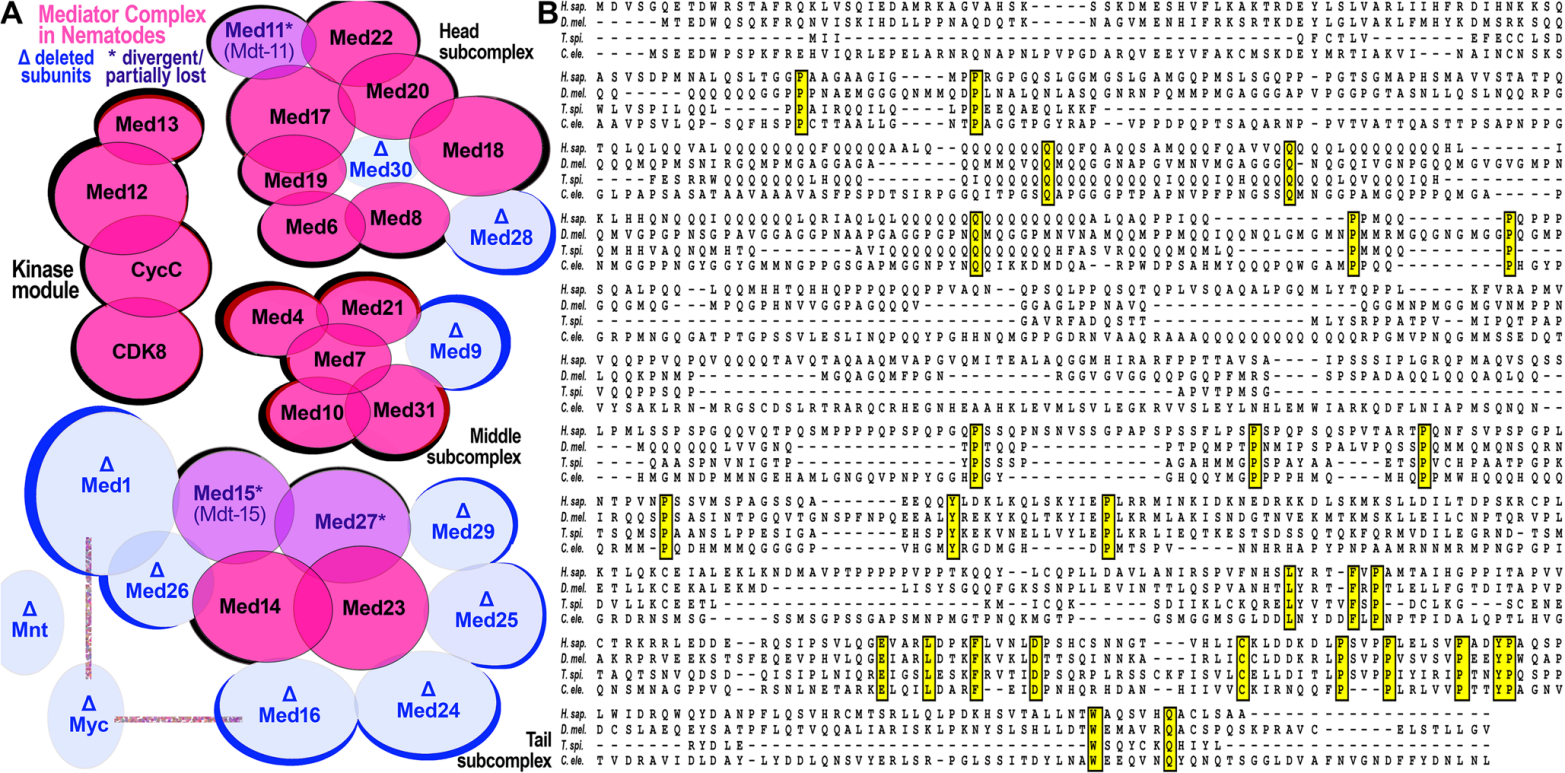
Reduction of Mediator complex accompanied loss of CIBLIN regulators in nematodes. **a** The head, middle, and tail subcomplexes, as well as the kinase module of Mediator is shown, along with the subunits that are not detectable in nematode genomes (specifically the genomes for species shown in Fig. 1a). The undetectable subunits, which are likely lost or else under relaxed selection and fast-evolving are indicated in *blue with a delta symbol* (“deleted”). Conserved subunits are indicated in *fuchsia*. Subunits in *purple* are putatively present as extremely divergent forms and have been given suggestive names Mdt-15 and Mdt-11. Med27 was only detected in the enoplian species of *Trichinella*. Human Myc is known to physically contact human Med1 and Med16 (*vertical and horizontal lines*, from Fig. 2a). **b** An alignment of the Med15 protein from human (H. sap.), fly (D. mel.), and the nematode *Trichinella* (T. spi.) and Mdt-15 from *C. elegans* (C. ele.), which is most likely Med-15, is highlighted here to make several points about the threshold sensitivity of the CIBLIN repertoire. The EnsemblCompara pipelines are able to make the call for Med15 in *Trichinella* (Ensembl Metazoa EnsemblCompara) but not in *C. elegans* (both Metazoan Ensembl Compara and the main Ensembl Genes 78 computation). Med15 protein sequence does not feature any major domains and at no place is there more than a single amino acid residue conserved twice in a row in all four species. Insertions and deletions predominate, and few residues are conserved across all taxa (*yellow highlight*). Med15/Mdt15 is not a CIBLIN gene because of its detection in *Trichinella*

We find that nearly every major DNA binding domain is evenly represented among the 101 CIBLIN regulators including zinc finger domains (ZF), bHLH, bZIP, homeodomain (HD), rel homology domain (RHD), and nuclear hormone receptors (NHR) (Fig. 2a). Importantly, Myc is ranked as having the most interactions for any regulator outside of the lost Mediator subunits for this set of top ranked interactions. Myc also has the only physical contacts with Mediator subunits. Figure 3 summarizes the Mediator complex (red subunits in Fig. 3) in nematodes in relation to their undetectable CIBLIN subunits (blue subunits in Fig. 3). It should be noted however that the Mediator complex in nematodes has not been biochemically purified and that many of the putative subunits are based solely on the best alignments, many of which are admittedly weak and the basis for using the “Mdt” names in *C. elegans* and “Med” names in all other organisms including yeast (Blazek et al. 2005). Many alignments of these putative homologs with bona fide Mediator subunits are poor and based on short peptide sequences embedded in otherwise flexible or intrinsically disordered protein regions (Taubert et al. 2006). A good example is Med15/Mdt-15, which is not a CIBLIN gene because it is detected in *Trichinella* despite the lack of a well-defined protein domain and the presence of many insertions (see Fig. 3b). Putative Mediator orthologs are proposed to exist as Mdt1.1 and Mdt1.2 for Med1, Mdt-24 (LIN-25) for Med24, Mdt29 for Med19, Mdt-9 for Med9, Mdt-28 for Med28, and Mdt-30 (PQN-30) for Med30 (Grants et al. 2015). However, no orthologs have been have been found for the “lost” Mediator subunits (Med16 and Med25) and at least these have been proposed to be absent (Grants et al. 2015). Nonetheless, Fig. 3 accurately reflects Mediator subunits that can be confidently assigned for nematodes versus those that are definitively lost or for which extremely divergent homologs have been tentatively proposed based on sequence alignment and some genetic studies of their function.

When we look at co-expression of CIBLIN regulators, we find that over one-half of the 101 human CIBLIN regulators are tightly co-regulated with *MYC*, *MYCN*, *MYCL*, or *MNT* based on hundreds of transcriptomics studies (Fig. 2b). The vast majority of these are co-expressed with *MYC* to a greater degree than with *MNT* or the other *MYC* paralogs *MYCN* or *MYCL* (Fig. 2b). We then inspected which particular transcriptomic studies were responsible for the correlations in the co-expression network of Fig. 2b and found that the vast majority (67 %) were from transcriptomic studies of diverse cancer types or cancer-related experimental designs (Supplementary Table S5).

Altogether, we see that Myc can be placed within an interacting network of lost CIBLIN genes regulating cell proliferation, differentiation, and apoptosis. A perfect example of other pleotropic proto-oncogenes connected to Myc are the genes in the proto-oncogenic *fos* family important in cell transformation (*FOS*, *FOSB*, *FOSL1*, and *FOSL2* in Fig. 2a, b) (Durchdewald et al. 2009). We thus conclude that the loss of Myc in nematodes can be understood as the loss of a complex gene network functioning in surveillance and control of cell proliferation during bulk organogenesis.

### The set of CIBLIN genes overlaps significantly with gene hits from MycSL screens

Cells from many cancers overexpress Myc, and this basic cancer signature has been exploited to identify synthetic lethal interactors of hyperactive Myc levels relative to wild-type Myc levels with shRNA/siRNA libraries (Cermelli et al. 2014; Kessler et al. 2012; Liu et al. 2012; Toyoshima et al. 2012). If the pleiotropic Myc regulator is critical to establishing states of gene expression conducive to cellular proliferation, some CIBLIN genes might be co-expressed with Myc and also turn up as MycSL hits. Alternatively, little overlap between CIBLIN and MycSL genes might be helpful for thinking about the coverage and specificity of such screens and the relative balance of proto-oncogenes versus tumor suppressors identified.

To determine whether the CIBLIN repertoire includes MycSL genes, we looked for overlap with the 397 MycSL hits from a screen in human mammary epithelial cells (HMECs) (Kessler et al. 2012), 11 MycSL kinome hits from an HMEC screen (Liu et al. 2012), and 101 MycSL druggable hits from a screen in human foreskin fibroblasts (HFFs) (Toyoshima et al. 2012). The latter two MycSL screens each have only one gene in common with the first screen (*GSK3B* or *BRD4*, respectively) suggesting that the experimental design is sensitive to cell-type (HMECs vs HFFs), to different ectopic levels of Myc (inducible Myc-ER vs retrovirus expressed Myc), and/or to the efficacy of knockdown method (shRNA vs siRNA libraries).

**Fig. 4:**
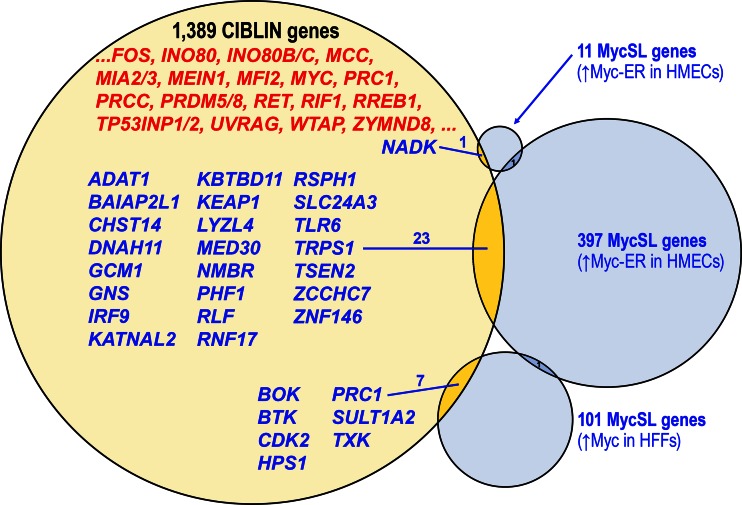
Human CIBLIN genes include Myc synthetic lethal hits from several screens. A Venn diagram of overlap between human CIBLIN genes and Myc synthetic lethal (MycSL) hits identified by screening small hairpin RNA (shRNA) or siRNA libraries. The list of 1389 human CIBLIN genes were cross-checked with the 11 MycSL kinome hits in a screen using human mammary epithelial cells (HMECs), 397 MycSL hits found in an HMEC screen, and 101 MycSL hits from a screen in human foreskin fibroblasts (HFFs) (Kessler et al. 2012; Liu et al. 2012; Toyoshima et al. 2012). The first two studies produced ectopic Myc using an inducible Myc-ER fusion, while the third screen used a retroviral vector to drive expression of ectopic levels of Myc. Thirty-one or ~6.1 % of human CIBLIN genes were found to be MycSL hits in one of the three MycSL screens as indicated. Thus, there is more overlap between the CIBLIN genes and any one MycSL screen than overlap between the MycSL screens themselves. In addition, the list of human CIBLIN genes include many important factors connected to cancer progression but not directly connected to Myc-related pathways (list of genes in red includes a small sample of relevant genes not listed in other figures). See also Supplementary Table S6 for a breakdown of genes

We find that 31 CIBLIN genes (or 6.1 % of 1389 CIBLIN genes) are MycSL hits from all three screens (Fig. 4, and Supplementary Table S6). Highlighting the unique nature of the nematode gene reduction, six of these 31 genes are also present in yeast: *CDK2*, *KATNAL2*, *TRPS1*, *TSEN2*, *ZCCHC7*, and *ZNF146* [We note that while there is a gene “named” *cdk-2* in *C. elegans*, *cdk-2* orthologs are identified computationally only in other nematodes. Furthermore, this nematode *cdk-2* gene is understood to be the closest gene to CDK2 other than nematode *cdk-1*/*CDK1*, which appears to be more similar to bilaterian CDK-2 than nematode *cdk-2* (Liu and Kipreos 2000)].

In addition to containing MYC and many MycSL hits from different screens, the CIBLIN list contains an astonishing number of known proto-oncogenes first discovered for their roles in cancer and/or cellular transformation. These include *mutated in colorectal cancers* (*MCC*) (Kinzler et al. 1991); migration and invasion enhancer 1 (*MEIN1*) (Evans et al. 2006); melanoma inhibitory activity *MIA2/3/CTAGE* family members (Blesch et al. 1994); papillary renal cell carcinoma, translocation-associated (*PRCC*) (Sidhar et al. 1996); many genes connected to *ras* proto-oncogenic signaling such as *RREB1*, ras responsive element binding protein 1 (Thiagalingam et al. 1996); the *RET* proto-oncogene (Takahashi et al. 1985); UV radiation resistance associated (*UVRAG*) (Iida et al. 2000); Wilms tumor 1 associated protein (*WTAP*) (Gessler et al. 1990; Pritchard-Jones et al. 1990); and many others. In summary, the CIBLIN repertoire is enriched for many proto-oncogenic developmental regulators of cell proliferation, cell migration, ECM remodelers, and stem cell maintenance. This suggests that the CIBLIN set of genes should be taken seriously as a linked set of developmental regulators controlling bulk cellular quantity during organogenesis (Supplementary Tables S1, S2, S3, and S4).

What these analyses do not yet address is whether the CIBLIN gene list is significantly enriched or depleted for tumor suppressors as well. At first glance, it seems that these types of genes are not as prominent as homologs of human proto-oncogenes. It is possible that this reflects a fundamental difference between the molecular functions of such genes. Tumor suppressors that implement check points or damage surveillance of various types might still be required in nematode cells, and many such genes (e.g., p53, PTEN) have been studied for their roles in DNA damage checkpoints and apoptosis (Derry et al. 2001; Liu and Chin-Sang 2015; Schumacher et al. 2001). Indeed, both apoptosis and autophagy are required throughout *C. elegans* development (Borsos et al. 2011).

## Discussion

Here, we identified a maximum of 839 orthology groups (orthologs that may have duplicated in any one lineage) that are CIBLIN genes using methods similar to the identification of 25 CIELIM genes *conserved in eukaryotes/lost in metazoans* (Erives and Fassler 2015). The majority of CIELIM genes lost in the stem-metazoan lineage pertain to the gradual reduction of biosynthetic pathways, which explains the requirement in animals for dietary sources of essential amino acids and many vitamin co-factors. In contrast, CIBLIN genes correspond to developmental regulators of cell proliferation, cell migration and ECM remodeling, apoptosis, stem cell maintenance, cell cycle checkpoints, and Mediator co-activator subunits. The preponderance of genes related to cell migration likely indicates that these functions are as equally impacted by the evolutionary reduction in body size as are cell proliferation regulators. These are interesting losses given the substantial number of canonical developmental pathways (e.g., EGF, FGF, Hedgehog, Notch, and Wnt pathways) maintained in nematodes (Kolotuev et al. 2009; Minor et al. 2013; Schmid and Hajnal 2015). However, even components of the Hippo pathway, which is intimately connected to regulation of organ size, are mostly conserved (Yang and Hata 2013).

The answer to whether some of the CIBLIN genes are actually present in nematodes and only fast evolving and undetectable by the methods used here does not address why cell proliferation and cell migration genetic functions are predominantly enriched in the set of genes presented here. For this reason, we propose that the CIBLIN genes were under relaxed selection given nematode developmental evolution and that the majority were eventually lost early in nematode evolution. Below, we explain why CIBLIN gene deletions might actually have been under positive selection.

We speculated previously that the evolution of gastrulation and endodermal tissues in Metazoa might be intricately linked with the loss of the CIELIM genes. Genes required for embryonic development and patterning of a multicellular high-throughput filter feeding organism could have been favored over genes encoding enzymes for producing molecules that could now be derived as nutrients from dietary sources via the endodermal tissues of gastrulation (Erives and Fassler 2015). While it is difficult to disentangle the causes and effects of CIELIM gene losses and the evolutionary adaptations of proto-animals, a brief discussion on the loss of CIBLIN genes in nematodes may be clinically relevant in one other way besides cancer progression.

The immediate explanation for the loss of the CIBLIN genes is that they were no longer required by these small-bodied animals to regulate large populations of somatic cells. These developmental genetic functions might have served at the level of tissue induction or homeostatic control of organ size maintenance. Alternatively, these genetic functions might have served to canalize developmental processes, a role which may have been rendered superfluous by the evolution of strict cell fate determinative mechanisms based on well-defined cell lineages.

Many nematodes (*Brugia*, *Loa*, *Onchocerca*) are filarial parasites afflicting humans and other mammals via transmission in dipterans (black flies and mosquitoes). *Trichinella* is a nematode parasite that causes trichinosis in humans, but members of this ancient clade affect all vertebrate groups and many have complex life cycles through multiple hosts. Furthermore, parasitic nematodes are known to exert effective immunomodulation of their hosts via excretory-secretory molecules (Hewitson et al. 2009; Jex et al. 2014). Nonetheless, most if not all non-parasitic (free-living) nematodes are also closely associated with specific animals (Kiontke and Sudhaus 2006; Schulte 1989). *Pristionchus* is associated with scarab beetles. *Caenorhabditis remanei*, *C. elegans*, and *C. briggsae* are found together and are thought to use snails, slugs, millipedes, mites, and pill bugs to transport the dormant dauer stage. *C. japonica* is associated specifically with shield bugs and stink bugs. In this context, it is interesting that many of the lost proteins are transcriptional activators and co-activators with interaction domains that allow them to aggregate into large regulatory complexes. Thus, an interesting question is whether loss of such proteins ever facilitated commensalism or parasitism because of a reduced antigenic footprint.

We conclude by pointing out that the CIBLIN genes might constitute a high priority genomic platform to be studied together. In other words, it may be useful to think about their joint loss and how they work together to control cell proliferation. While the normal roles of such genes can be studied in flies, mice, and humans, studies in nematodes might be useful for highlighting how conserved developmental gene regulatory networks operate in the absence of CIBLIN gene regulatory networks (Brown et al. 2008).

## Electronic supplementary material

Table S1 (XLSX 319 kb)

Table S2 (XLSX 71 kb)

Table S3 (XLSX 188 kb)

Table S4 (XLSX 152 kb)

Table S5 (PDF 36 kb)

Table S6 (PDF 37 kb)
