# Supplementary material for: Genes conserved in bilaterians but jointly lost with Myc during nematode evolution are enriched in cell proliferation and cell migration functions
Source: Dev Genes Evol. 2015 Jul 15;225(5):259–73. doi: 10.1007/s00427-015-0508-1 (PMC4568025; doi:10.1007/s00427-015-0508-1)
Supplement: Supplementary file 5 — (PDF 36 kb) [file 427_2015_508_MOESM5_ESM.pdf]

Supplementary Table S5. CIBLIN regulators are co-expressed with *MYC* in cancer transcriptomes.

Study	Weight	Reference	Num. of interactions* (Source)	Nature of transcriptome study
Wang-Maris-2006	7.04%	Integrative genomics identifies distinct molecular classes of neuroblastoma and shows that multiple genes are targeted by regional alterations in DNA copy number. (Wang et al. 2006)	264,234 (NCBI GEO)	cancer, neuroblastoma
Ramaswamy-Golub-2001	6.60%	Multiclass cancer diagnosis using tumor gene expression signatures. (Ramaswamy et al. 2001)	270,142 (Supp. mat.)	cancer
Bild-Nevins-2006 B	6.34%	Oncogenic pathway signatures in human cancers as a guide to targeted therapies. (Bild et al. 2006)	282,582 (NCBI GEO)	cultured cells, cancer, epithelial cells, cell line, breast cancer
Salaverria-Siebert-2011	6.31%	Translocations activating IRF4 identify a subtype of germinal center-derived B-cell lymphoma affecting predominantly children and young adults. (Salaverria et al. 2011)	514,070 (NCBI GEO)	cancer
Mallon-McKay-2013	5.80%	StemCellDB: the human pluripotent stem cell database at the National Institutes of Health. (Mallon et al. 2013)	567,140 (NCBI GEO)	cultured cells, stem cells, cell line
Burington-Shaughnessy-2008	5.75%	Tumor cell gene expression changes following short-term in vivo exposure to single agent chemotherapeutics are related to survival in multiple myeloma. (Burington et al. 2008)	293,587 (NCBI GEO)	time series, cancer, chemotherapy
Bahr-Bowler-2013	5.62%	Peripheral blood mononuclear cell gene expression in chronic obstructive pulmonary disease. (Bahr et al. 2013)	278,447 (NCBI GEO)	pulmonary disease, inflammatory response
Alizadeh-Staudt-2000	5.52%	Distinct types of diffuse large B-cell lymphoma identified by gene expression profiling. (Alizadeh et al. 2000)	88,888 (Supp. mat.)	cultured cells, cancer
Rieger-Chu-2004	5.01%	Toxicity from radiation therapy associated with abnormal transcriptional responses to DNA damage.(Rieger et al. 2004)	259,055 (NCBI GEO)	cultured cells, cell line, DNA damage
Roth-Zlotnik-2006	4.94%	Gene expression analyses reveal molecular relationships among 20 regions of the human CNS. (Roth et al. 2006)	666,614 (NCBI GEO)	developmental study
Innocenti-Brown-2011	4.67%	Identification, replication, and functional fine-mapping of expression quantitative trait loci in primary human liver tissue. (Innocenti et al. 2011)	579,361 (NCBI GEO)	epithelial cells, cultured cells, cell line, liver cancer
Smirnov-Cheung-2009	4.66%	Genetic analysis of radiation-induced changes in human gene expression. (Smirnov et al. 2009)	463,390 (NCBI GEO)	cultured cells, cell line, DNA damage
Gysin-McMahon-2012	4.61%	Analysis of mRNA profiles after MEK1/2 inhibition in human pancreatic cancer cell lines reveals pathways involved in drug sensitivity.(Gysin et al. 2012)	388,454 (NCBI GEO)	cell proliferation, cultured cells, cell line, cancer
Kang-Willman-2010	4.37%	Gene expression classifiers for relapse-free survival and minimal residual disease improve risk classification and outcome prediction in pediatric B-precursor acute lymphoblastic leukemia. (Kang et al. 2010)	656,632 (NCBI GEO)	lymphoma cancer
Arijs-Rutgeerts-2009	4.18%	Mucosal gene expression of antimicrobial peptides in inflammatory bowel disease before and after first infliximab treatment. (Arijs et al. 2009)	653,194 (NCBI GEO)	immune system
Boldrick-Relman-2002	4.17%	Stereotyped and specific gene expression programs in human innate immune responses to bacteria. (Boldrick et al. 2002)	108,543 (Supp. mat.)	immune system
Wu-Garvey-2007	3.67%	The effect of insulin on expression of genes and biochemical pathways in human skeletal muscle. (Wu et al. 2007)	260,762 (NCBI GEO)	muscle cultured cells, stress response
Chen-Brown-2002	3.63%	Gene expression patterns in human liver cancers. (Chen et al. 2002)	275,649 (Supp. mat.)	cell proliferation. liver cancer
Cheok-Evans-2003	3.60%	Treatment-specific changes in gene expression discriminate in vivo drug response in human leukemia cells. (Cheok et al. 2003)	263,940 (NCBI GEO)	chemotherapy cultured cells lymphoma cancer
Perou-Botstein-1999	3.50%	Distinctive gene expression patterns in human mammary epithelial cells and breast cancers. (Perou et al. 1999)	62,886 (Supp. mat.)	cultured cells, cancer epithelial cells, breast stromal cells, breast cancer

* Number of interactions subjected to Pearson correlation

Studies which were the most significantly associated with co-expressed CIBLIN transcriptional regulators are predominantly associated with cancer transcriptomes (highlighted in yellow), which collectively contributed 67.74% of the CIBLIN correlation. This was followed by DNA damage (highlighted in red), and immune system (highlighted in blue).
