# Supplementary material for: Genes conserved in bilaterians but jointly lost with Myc during nematode evolution are enriched in cell proliferation and cell migration functions
Source: Dev Genes Evol. 2015 Jul 15;225(5):259–73. doi: 10.1007/s00427-015-0508-1 (PMC4568025; doi:10.1007/s00427-015-0508-1)
Supplement: Supplementary file 6 — (PDF 37 kb) [file 427_2015_508_MOESM6_ESM.pdf]

Supplementary Table S6. CIBLIN genes outnumber Myc synthetic lethal (MycSL) hits

Human gene name	971 single copy mammal. CIBLIN genes	187 non-single copy CIBLIN genes*	397 MycSL genes, Kessler (2012)	101 MycSL genes, Toyoshima (2012)	Gene description [Source: HGNC]
ADAT1	X		X		adenosine deaminase, tRNA-specific 1
BAIAP2L1	X		X		BAI1-associated protein 2-like 1
BOK	X			X	BCL2-related ovarian killer
BTK	X			X	Bruton agammaglobulinemia tyrosine kinase
CHST14	X		X		carbohydrate (N-acetylgalactosamine 4-0) sulfotransferase 14
DNAH11	X		X		dynein, axonemal, heavy chain 11
GCM1	X		X		glial cells missing homolog 1 (Drosophila)
GNS		X	X		glucosamine (N-acetyl)-6-sulfatase
HPS1	X			X	Hermansky-Pudlak syndrome 1
IRF9		X	X		interferon regulatory factor 9
KBTBD11	X		X		kelch repeat and BTB (POZ) domain containing 11
KEAP1	X		X		kelch-like ECH-associated protein 1
LYZL4	X		X		lysozyme-like 4
MED30	X		X		mediator complex subunit 30
NMBR	X		X		neuromedin B receptor
PHF1	X		X		PHD finger protein 1
PRC1	X			X	protein regulator of cytokinesis 1
RLF	X		X		rearranged L-myc fusion
RNF17	X		X		ring finger protein 17
RSPH1	X		X		radial spoke head 1 homolog (Chlamydomonas)
SLC24A3	X		X		solute carrier family 24 (Na/K/Ca exchanger), 3
SULT1A2	X			X	sulfotransferase fam., cytosolic, 1A, phenol-pref, 2
TLR6		X	X		toll-like receptor 6
TXK	X			X	TXK tyrosine kinase
ZNF146	X		X		zinc finger protein 146

* CIBLIN genes duplicated after mammalian diversification (human, mouse, rat).
